# Reply to Yu et al.: Datasets, human judges, and future directions for evaluating AI–AI bias

**DOI:** 10.1073/pnas.2522651122

**Published:** 2025-09-26

**Authors:** Jan Kulveit, Walter Laurito, Tomáš Gavenčiak, Peli Grietzer, Benjamin Davis, Ada Böhm

**Affiliations:** ^a^Alignment of Complex Systems Research Group, Center for Theoretical Studies, Charles University, Prague 11000, Czech Republic; ^b^Information Process Engineering, Forschungszentrum Informatik, Karlsruhe 76131, Germany; ^c^Arb Research, Prague 11636, Czech Republic; ^d^Private address, Andover, MA 04216

We appreciate the thought-provoking commentary by Yu et al. (1) on our work (2).

Before responding to the more conceptual questions about identity, it is important to address a possible misunderstanding about the datasets used in the paper and the comparisons made.

Aiming for ecological validity, the human-written datasets we used were i) actual product descriptions from e-commerce websites, ii) actual abstracts of research papers, and iii) plot summaries extracted from Wikipedia articles. The first two datasets are typically expert-written; e-commerce sellers often hire professionals to create product descriptions, and academic authors invest substantial effort in crafting abstracts. While Wikipedia is written by a large group of volunteer editors, it is often used or displayed by various other sites, making it a sensible choice as the human alternative.

Exactly because of the possibility that LLMs could just use effective communication patterns aligned with humans, we had a group of research assistants evaluate the human- and LLM-written presentations as well. Crucially, we operationalize AI–AI bias as the extent to which LLM preference for LLM-generated text exceeds human preference. The results in our work (Fig. 1; Tables 4 and 5) (2) show a gap between human and LLM choices so the LLM choices are not generally “aligned,” at least as far as we consider the opinions of the research assistants to be the ground truth. Nonetheless, we agree that expanding the human evaluations to larger and more diverse cohorts is warranted; we encourage future work to do so, as noted in the Discussion. If we discard the assumption that performance should be optimized for communication to humans, the meaning of “effective” loses its grounding. For example, the main result could be characterized as LLMs being more effective in communication to AI agents than humans are, but this highlights the systemic risk we sought to investigate rather than negating the observed bias relative to human standards.

Regarding the more thorny question of whether the results are best understood using the frame of “identity”-as we note in the paper, we mostly suggest the frame of identity is a useful way to understand the experimental design and the implications. Alternatively, the results could be described as evaluated AI agents reacting to “stylistic regularities” characteristic to other AI written texts, and in some sort of “halo effect” this reaction leading to preference for options where the description is in this characteristic “AI style.” This still seems epistemically defective-as the style of the prose is inappropriately treated as evidence of the superiority of the presented good-and has the same negative impacts on humans.

We wholeheartedly agree on the need for both further empirical work involving diverse human evaluators, and work to disentangle the possible causes of the effect.
